# Mining 3D Patterns from Gene Expression Temporal Data: A New Tricluster Evaluation Measure

**DOI:** 10.1155/2014/624371

**Published:** 2014-03-31

**Authors:** David Gutiérrez-Avilés, Cristina Rubio-Escudero

**Affiliations:** Department of Computer Science, University of Seville, Avenida Reina Mercedes s/n, 41012 Seville, Spain

## Abstract

Microarrays have revolutionized biotechnological research. The analysis of new data generated represents a computational challenge due to the characteristics of these data. Clustering techniques are applied to create groups of genes that exhibit a similar behavior. Biclustering emerges as a valuable tool for microarray data analysis since it relaxes the constraints for grouping, allowing genes to be evaluated only under a subset of the conditions. However, if a third dimension appears in the data, triclustering is the appropriate tool for the analysis. This occurs in longitudinal experiments in which the genes are evaluated under conditions at several time points. All clustering, biclustering, and triclustering techniques guide their search for solutions by a measure that evaluates the quality of clusters. We present an evaluation measure for triclusters called Mean Square Residue 3D. This measure is based on the classic biclustering measure Mean Square Residue. Mean Square Residue 3D has been applied to both synthetic and real data and it has proved to be capable of extracting groups of genes with homogeneous patterns in subsets of conditions and times, and these groups have shown a high correlation level and they are also related to their functional annotations extracted from the Gene Ontology project.

## 1. Introduction

The use of high throughput processing techniques has revolutionized the technological research and has exponentially increased the amount of data available [1]. Particularly, microarrays have revolutionized biological research by their ability to monitor changes in RNA concentration in thousands of genes simultaneously [2].

A common practice when analyzing gene expression data is to apply clustering techniques, creating groups of genes that exhibit similar expression patterns [3]. These clusters are interesting because it is considered that genes with similar behavior patterns can be involved in similar regulatory processes [4]. Although in theory there is a big step from correlation to functional similarity of genes, several articles indicate that this relation exists [5].

Traditional clustering algorithms work on the whole space of data dimensions examining each gene in the dataset under all conditions tested. However, the activity of genes could only appear under a particular set of experimental conditions, exhibiting local patterns. Discovering these local patterns can be the key to discover gene pathways, which could be hard to discover in other ways. For this reason, the paradigm of clustering techniques must change to methods that allow local pattern discovery in gene expression data [6].

Biclustering [7] addresses this problem by relaxing the conditions and by allowing assessment only under a subset of the conditions of the experiment, and it has proved to be successful in finding gene patterns [8]. However, if the time condition is added to the dataset clustering, and biclustering result insufficient. There is a lot of interest in temporal experiments because they allow an in-depth analysis of molecular processes in which the time evolution is important, for example cell cycles, development at the molecular level or evolution of diseases [9]. In this sense, triclustering appears as a valuable tool since it allows for the assessment of genes under a subset to the conditions of the experiment and under a subset of times.

All clustering, biclustering, and triclustering techniques guide their search for solutions by a measure that evaluates the quality of clusters [10]. In this work we propose an evaluation measure for triclusters called Mean Square Residue 3D (MSR_3D_). This measure is based on a classic biclustering measure presented by Cheng and Church in [11] called Mean Square Residue (MSR). MSR measures the homogeneity of a bicluster in the relation of each value in the bicluster with the average value for all genes in the bicluster, average of all conditions, and average of all genes and conditions in the bicluster. A perfect score would be zero, which represents a constant bicluster of elements of a single value.

Our proposal, MSR_3D_, is an adaptation of MSR to the three-dimensional space, so that a third factor, in this case time, can be taken into account. MSR_3D_ measures the homogeneity of a tricluster in the relation of each value of the tricluster, with the average of all genes, average of all conditions, average of all times, average of all genes and conditions, average of all genes and times, average of all conditions and times, and average of all genes, conditions, and times in the tricluster. As for MSR, a perfect score would be zero, which represents a constant tricluster of elements of a single value.

MSR_3D_ has been applied as an evaluation measure along with the TriGen (*Tri*clustering-*Gen*etic based) algorithm presented in [12]. TriGen is an algorithm based on evolutionary heuristic, genetic algorithms. Many heuristic approaches have been proposed both for biclustering and triclustering algorithms [13, 14], due to the NP hard nature of the problem [15].

We show the results obtained from applying the TriGen algorithm along with the MSR_3D_ measure to a synthetic dataset and four real experiments datasets: the yeast cell cycle regulated genes [16], mouse degeneration of retinal cells [17], mouse ectopic bHLH transcription factor expression Mesogenin1 effect on embryoid bodies [17], and human Transcription factor oncogene OTX2 silencing effect on D425 medulloblastoma cell line [17].

The results have been validated by analyzing the correlation among the genes, conditions, and times in each tricluster using two different correlation measures: Pearson and Filon [18] and Spearman [19]. Besides this, we have provided functional annotations for the genes extracted from the Gene Ontology project [20].

The rest of the paper is structured as follows. A review of the latest related works can be found in Section 2.  Section 3 describes the methodology of the MSR and MSR_3D_ measures as well as a brief description of the TriGen algorithm. In Section 4 we show the results of applying TriGen to the synthetic and real datasets.  Section 5 shows the conclusions.

## 2. State of the Art

This section is to provide a general overview of recent works in the field of gene expression temporal data. In particular, for those works related to the application of triclustering, we focus on the measures applied to evaluate the triclusters.

In 2005, Zhao and Zaki [21] introduced the triCluster algorithm to extract patterns in 3D gene expression data. They presented a measure to assess triclusters' quality based on the symmetry property. This allows a very efficient cluster mining since clusters are searched over the dimensions with the least cardinality. The triclusters have to fulfill some requirements such as being maximal; that is, no tricluster in the set of solutions is totally included in another tricluster in the set of solutions; the ratio of every pair of columns in the tricluster is delimited by a given *ϵ*; the maximum volume of the tricluster is determined by the relation among *δ* ^*x*^, *δ* ^*y*^, and *δ* ^*z*^ for gene, condition, and time dimensions,respectively; and the minimum volume for the tricluster is also controlled. An extended and generalized version of this proposal, g-triCluster, was published one year later [22]. The authors claimed that the symmetry property is not suitable for all patterns present in biological data and propose the Spearman rank correlation [19] as a more appropriate tricluster evaluation measure.

An evolutionary computation proposal was made in [23]. The fitness function defined is a multiobjective measure which tries to optimize three conflicting objectives: clusters size, homogeneity, and gene-dimension variance of the 3D cluster.

LagMiner was introduced in [24] to find time-lagged 3D clusters, which allows in turn finding regulatory relationships among genes. It is based on a novel 3D cluster model called *S* ^2^ *D* ^3^ Cluster. They evaluated their triclusters on homogeneity, regulation, minimum gene number, sample subspace size, and time periods length.

Wang et al. [25] proposed a new algorithm called ts-cluster basing their definition for coherent triclusters also on finding regulatory relationships among genes. For that purpose, time shifting is also considered among time points in the evaluated triclusters.

A new strategy to mine 3D clusters in real-valued data was introduced in [26]. The authors defined the Correlated 3D Subspace Clusters (CSCs) where the values in each cluster must have high cooccurrences and those cooccurrences are not by chance. They measure the clusters based on the correlation information measure, which takes into account both prerequisites. In particular, the authors were concerned about discovering subspaces with a significant number of items, one of the main problems typically found in tricluster-based approaches. At the same conference, another approach was presented focusing on the concept of Low-Variance 3-Cluster [27], which obeys the constraint of a low-variance distribution of cell values.

The work in [28] was focused on finding Temporal Dependency Association Rules, which relate patterns of behaviour among genes. The rules obtained are to represent regulated relations among genes.

Finally, a brief survey on triclustering applied to gene expression time series was published in 2011 [29].

## 3. Methodology

In this section we first describe what is triclustering in relation to biclustering, second we show the fundamentals of our proposal, the two dimensions MSR measure proposed by Cheng and Church [11] in order to assess the quality of biclusters grouping gene and conditions, and third we make a detailed description of our proposal, the three dimensions MSR measure (MSR_3D_) to assess the quality of triclusters which group gene, conditions, and the time dimension. Finally, we describe TriGen and the genetic algorithm where the (MSR_3D_) measure has been integrated to be tested.

### 3.1. Triclustering

Given a dataset containing information from gene expression data organized in rows/columns (genes as rows and conditions as columns), biclustering finds subgroups of genes and conditions where the genes exhibit highly correlated patterns of behavior for every condition [30].

A bicluster BC can be defined as a subset from a dataset *D* which contains information related to the behavior of some genes *G*_*D*_ under certain conditions *C*_*D*_. The tricluster TC is formally defined as TC = *G* × *C* where *G*⊆*G*_*D*_ and *C*⊆*C*_*D*_.

**Figure 1:**
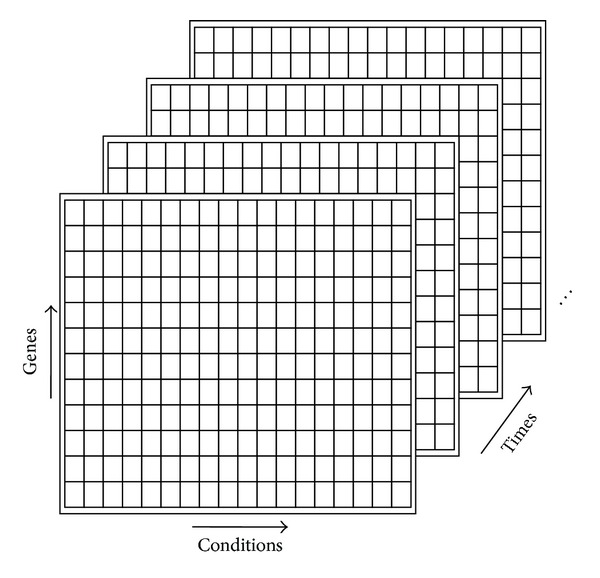
Tricluster representation.

Triclustering appears as an evolution of biclustering due to its capacity to mine gene expression datasets involving time as a third dimension and to find subgroups of genes, conditions, and times which exhibit highly correlated patterns of expression [12].  Figure 1 shows the structure of a tricluster, with genes as rows, conditions as columns, and time as depth.

A tricluster TC is as a subset from a dataset *D* which contains information related to the behavior of some genes *G*_*D*_ under conditions *C*_*D*_ at times *T*_*D*_. The tricluster TC is formally defined as TC = *G* × *C* × *T* where *G*⊆*G*_*D*_, *C*⊆*C*_*D*_, and *T*⊆*T*_*D*_.

### 3.2. Two-Dimension MSR

The Mean Squared Residue (MSR) was introduced by Cheng and Church in [11]. This measure was proposed to assess the quality of biclusters extracted from gene expression data based on biclusters' homogeneity. The formal definition can be seen in

(1) $$\begin{array}{c} \text{MSR}\left(\text{BC}\right)=\frac{\sum_{g\epsilon G,c\epsilon C}^{}r_{gc}^{2}}{\#G*\#C}, \end{array}$$

where *r*_*gc*_ can be defined as

(2) $$\begin{array}{c} r_{gc}=BC_{v}\left(g,c\right)-M_{G}\left(c\right)-M_{C}\left(g\right)-M_{GC}. \end{array}$$

Each of the terms of (1) and (2) are defined as follows:

- BC: bicluster being evaluated,
- *G*: subset of genes of BC,
- *C*: subset of conditions of BC,
- #*G*: number of genes in BC,
- #*C*: number of conditions in BC,
- BC_*v*_(*g*, *c*): expression level of a gene *g* under condition *c* in BC,
- *M*_*G*_(*c*): mean of the values of a condition *c* under all genes in BC,
- *M*_*C*_(*g*): mean of the values of a gene *g* under all conditions in BC,
- *M*_*GC*_: mean value of all values in BC.

**Figure 2:**
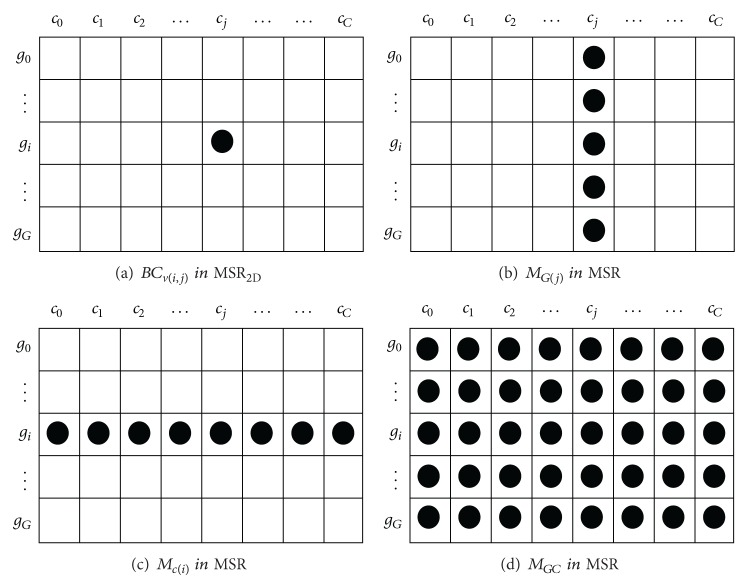
MSR members.

A graphical representation of the values involved in (2) can be seen in Figure 2. We can say that MSR measures the homogeneity for a given bicluster based on the difference of each individual gene expression BC_*v*(*i*,*j*)_ (see Figure 2(a)) with the average values of genes *M*_*G*(*j*)_ (see Figure 2(b)), conditions *M*_*C*(*i*)_ (see Figure 2(c)), and genes and conditions *M*_*GC*_ (see Figure 2(d)). The closer the value of MSR is to zero, the more homogeneous the bicluster is. This interpretation is the basis for the extension to three-dimension measure MSR_3D_ presented in the next section.

### 3.3. Three Dimensions MSR

Our proposal is an adaptation to three dimensions of MSR that measures the homogeneity of triclusters which contain subgroups of genes, conditions, and time points. We call this measure MSR_3D_. The formal definition can be seen in

(3) $$\begin{array}{c} \text{MS}{\text{R}}_{3\text{D}}\left(\text{TC}\right)=\frac{\sum_{g\epsilon G,c\epsilon C,t\epsilon T}^{}r_{gct}^{2}}{\#G*\#C*\#T}, \end{array}$$

where *r*_*gct*_ can be defined as

(4) rgct=TCv(g,c,t)+MCT(g)+MGT(c)+MGC(t)−MG(c,t)−MC(g,t)−MT(g,c)−MGCT.

Each of the members of (3) and (4) is defined as follows:

- TC: tricluster being evaluated,
- *G*: subset of genes from TC,
- *C*: subset of conditions from TC,
- *T*: subset of times from TC,
- #*G*: number of genes in TC,
- #*C*: number of conditions in TC,
- #*T*: number of times in TC,
- TC_*v*_(*g*, *c*, *t*): expression level of gene *g* under condition *c* at time *t* in TC,
- *M*_*CT*_(*g*): mean of all conditions at all times for a gene *g* in TC,
- *M*_*GT*_(*c*): mean of all genes at all times for a condition *c* in TC,
- *M*_*GC*_(*t*): mean of all genes under all conditions at time *t* in TC,
- *M*_*G*_(*c*, *t*): mean of the values of a condition *c* and a time *t* under all genes in TC,
- *M*_*C*_(*g*, *t*): mean of the values of a gene *g* and a time *t* under all conditions in TC,
- *M*_*T*_(*g*, *c*): mean of the values of a gene *g* and a condition *c* under all times in TC,
- *M*_*GCT*_: mean value of all values in TC.

**Figure 3:**
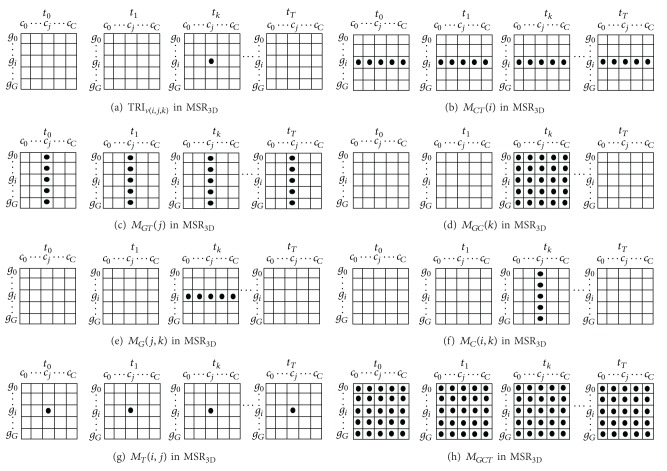
MSR_3D_ structural members.

A graphical representation of the values involved in (4) can be seen in Figure 3. We can say that MSR_3D_ measures the homogeneity for a given tricluster based on the difference of each individual gene expression TC_*v*_(*i*, *j*, *k*) (see Figure 3(a)), the mean of all conditions at all times for a gene *g* *M*_*CT*_(*g*) (see Figure 3(b)), the mean of all genes at all times for a condition *c* *M*_*GT*_(*c*) (see Figure 3(c)), the mean of all genes under all conditions at time *t* *M*_*GC*_(*t*) (see Figure 3(d)) with the mean of a condition *c* and a time *t* under all genes *M*_*G*_(*c*, *t*) (see Figure 3(e)), the mean of a gene *g* and a time *t* under all conditions *M*_*C*_(*g*, *t*) (see Figure 3(f)), the mean of a gene *g* and a condition *c* under all times *M*_*T*_(*g*, *c*) (see Figure 3(g)), and the mean value of all values in TC  *M*_*GCT*_ (see Figure 3(h)). The closer the value of MSR_3D_ is to zero, the more homogeneous the tricluster is. MSR_3D_ is capable of finding negatively correlated genes due to its formulation.

### 3.4. TriGen Algorithm

To test the effectiveness of MSR_3D_ we have included it as part of the TriGen (Triclustering-Genetic based) algorithm [12]. TriGen extracts triclusters from gene expression datasets where the time is also a component taken into account in the experiment. TriGen applies a bioinspired paradigm of an evolutionary heuristic, genetic algorithms, which mimics the process of natural selection by creating an initial population of individuals representing solutions which are crossed and mutated for a number of generations and the best individuals in the populations are finally selected. MSR_3D_ has been applied along with TriGen as a fitness function to assess the quality of the triclusters or solutions in the population.

**Figure 4:**
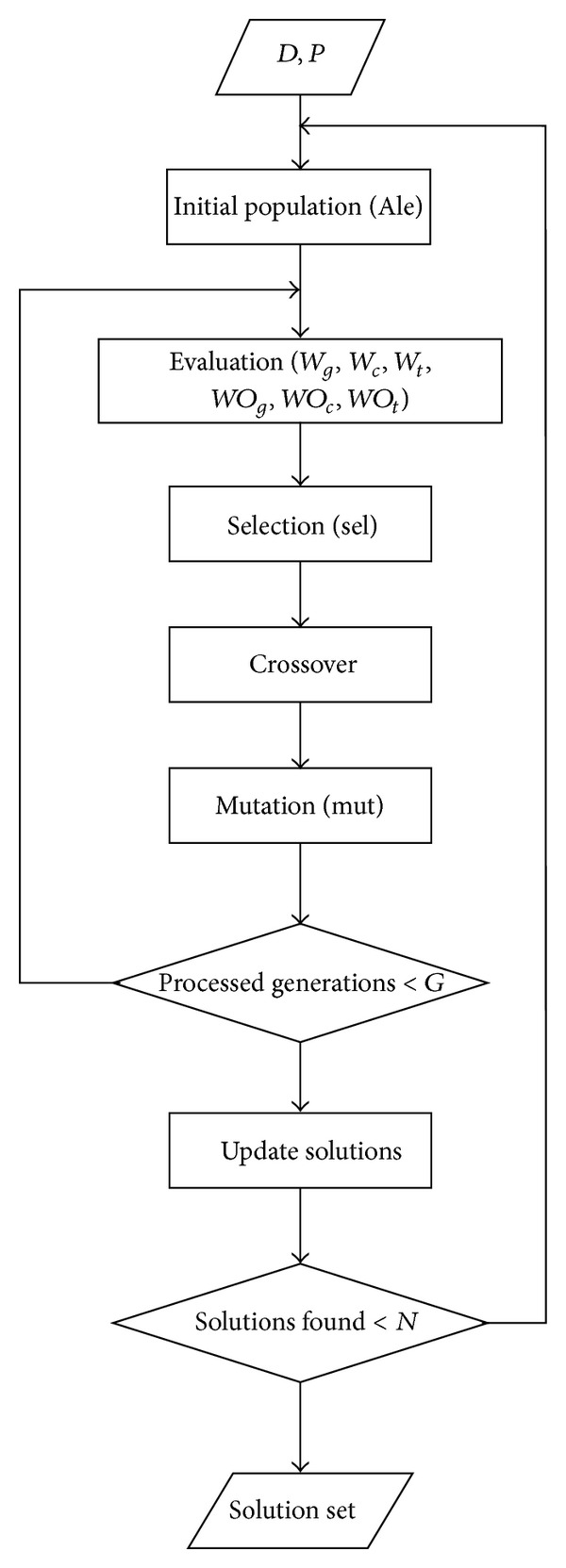
Flowchart for the TriGen algorithm.

The flowchart of the TriGen algorithm can be seen in Figure 4. In these subsections we are going to present the principal aspects of the algorithm including inputs, outputs, representation of individuals, and genetic operators.

#### 3.4.1. TriGen's Input

The* TriGen* algorithm takes two inputs:

- *D*: a dataset containing the gene expression values from an experiment containing genes *G*, experimental conditions *C*, and times *T*. Therefore, each cell [*i*, *j*, *k*] from *D* where *i* ∈ *G*, *j* ∈ *C*, and *k* ∈ *T* represents the expression level of the gene *i* under the experimental condition *j* at time *k*;
- *P*: set of parameters to execute the algorithm as described in Table 1. These parameters control the number of solutions or triclusters to find (*N*), the number of generations to execute (*G*), the number of individuals in the population (*I*), and the randomness factor which are generated within the initial population (*Ale*) as well as weights for the selection and mutation operators (sel y mut), weights to control the size of the triclusters (*w*_*g*_, *w*_*c*_, *w*_*t*_), and weights to control the overlap among solutions (*wo*_*g*_, *wo*_*c*_, *wo*_*t*_).

#### 3.4.2. TriGen's Output

The TriGen algorithm's output will be a set of *N* triclusters. Each tricluster is composed of a subset of genes *G*_*g*_, conditions *C*_*c*_, and times *T*_*t*_ from the input dataset *D*, with the best scores when evaluated under the MSR_3D_ measure.

#### 3.4.3. Codification of Individuals

Each individual in the evolutionary process of the* TriGen* algorithm represents a tricluster, that is, a subset of genes, experimental conditions, and time points. All genetic operators are applied to each individual in the population, in each of these three subsets. The genetic material is structured as follows. An individual, as mentioned above, is composed of three sequences of structures: one for the sequence of genes *G* from the input dataset *D*, one for the sequence of conditions *C*, and one sequence of time points *T*. These sequences are set up based on the input dataset; that is,

(5) $$\begin{array}{c} G=\langle g_{i_{1}},g_{i_{2}},\ldots ,g_{i_{B}}\rangle , \end{array}$$

where *B* is the number of genes listed in the input dataset, *i*_*j*_ < *i*_*j*+1_ for all genes, and 1 < *i*_*j*_ < *B*.

Analogously

(6) $$\begin{array}{c} C=\langle c_{i_{1}},c_{i_{2}},\ldots ,c_{i_{L}}\rangle , \end{array}$$

where *L* is the number of conditions listed in the input dataset, *i*_*j*_ < *i*_*j*+1_ for all conditions, and 1 < *i*_*j*_ < *L*.

Finally, *T* represents different time stamps or values of pairs gene condition at different times:

(7) $$\begin{array}{c} T=\langle t_{i_{1}},t_{i_{2}},\ldots ,t_{i_{M}}\rangle , \end{array}$$

where *M* is the number of samples measured over time and *t*_*i*_1__ < *t*_*i*_2__ < ⋯<*t*_*i*_*M*__.

**Figure 5:**
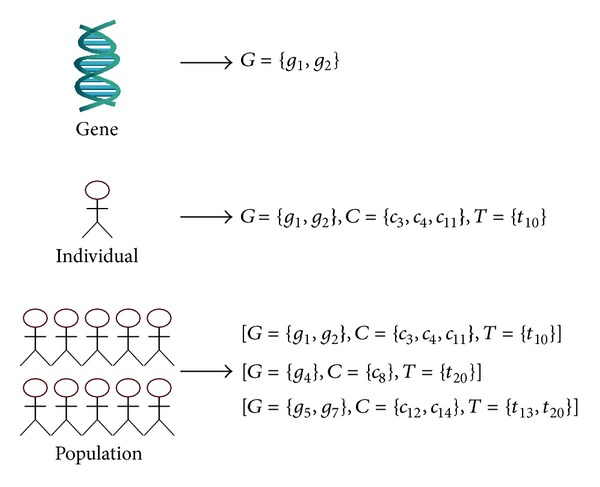
Genetic algorithm codification.

The algorithm's population is made up of several individuals, as depicted in Figure 5, where the individual codification has been represented.

#### 3.4.4. Initial Population

The initial population is generated attending to the *Ale* randomness parameter. An *Ale* percent of individuals are created at random by two methods: half of the individuals are purely randomly generated; this is a random subset of genes *G*_*g*_, conditions *C*_*c*_, and times *T*_*t*_ chosen from *D* and the other half is also randomly created but controlling that the values for the genes *G*_*g*_ are contiguous; the values for the conditions *C*_*c*_ are contiguous and the times *T*_*t*_ are contiguous as well. The rest of the individuals are created at random but taking into account the previously created individuals to control overlapping of solutions.

#### 3.4.5. Fitness Function

The proposed measure MSR_3D_ has been applied as part of the fitness function to evaluate the homogeneity of the triclusters in the population. MSR_3D_ has been combined with two other factors which measure the size of the triclusters and their overlap with previously found solutions.

Controlling the size of each of the dimensions of the triclusters might be a very important task since gene expression datasets are unbalanced on the three dimensions, with the number of genes counting in thousands and the number of conditions and times counting in tens. Therefore, the weights for the number of genes *w*_*g*_, of conditions *w*_*c*_, and times *w*_*t*_ control that the dimensions of the triclusters are balanced (e.g., if we increase *w*_*g*_, the algorithm considers that solutions with a high number of genes are better than those with low number of genes).

We also control the overlap among found solutions with the weights *wo*_*g*_, *wo*_*c*_, and *wo*_*t*_ for the overlap among genes, conditions, and times, respectively, (e.g., if we increase *wo*_*g*_, the algorithm considers that solutions with low level of overlap with the genes in previously found solutions are better than those with a high level of overlap).

Therefore, the fitness function can be formulated as seen in

(8) $$\begin{array}{c} \text{FF}\left(\text{TC}\right)=\text{MS}{\text{R}}_{3\text{D}}-\text{size}\;\text{control}-\text{overlap}\;\text{control}. \end{array}$$

#### 3.4.6. Selection Operator

This operator is implemented following the roulette wheel selection method [31]. The fitness level is used to associate a probability of selection with each individual of the population. This emulates the behavior of a roulette wheel in a casino. Usually a proportion of the wheel is assigned to each of the possible selections based on their fitness value. Then a random selection is made similar to how the roulette wheel is rotated. While candidates with a higher fitness will be less likely to be eliminated, there is still a chance that they are eliminated. There is a chance that some weaker solutions may survive the selection process, which is an advantage, as though a solution may be weak, it may include some component which could prove useful following the recombination process. The *Sel* parameter indicates how many individuals will pass to the next generation undergoing this method. The rest of the individuals up to complete the next population (*I* − #*Sel* *e* *c* *t* *e* *d* *individuals*) will be created based on the crossover operator.

#### 3.4.7. Crossover Operator

**Figure 6:**
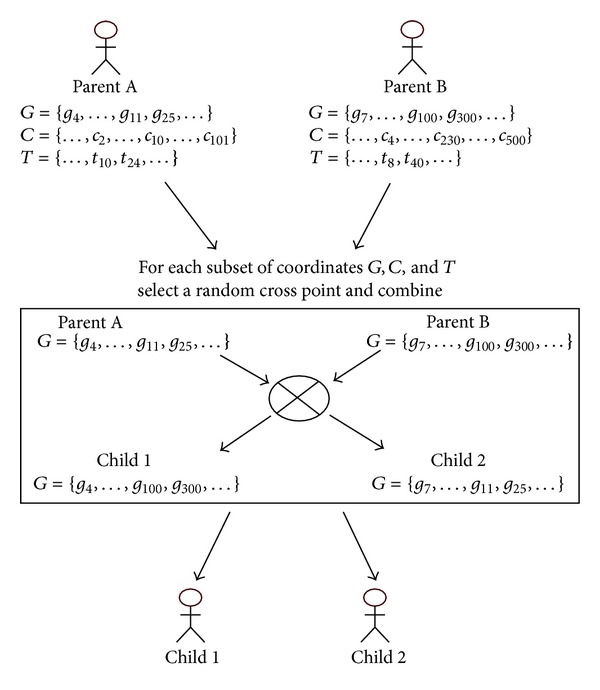
Representation of the crossover operator.

To complete the next generation, we create new individuals with this operator as follows: two individuals (parents, *A* and *B*) are combined to create two new individuals (offspring, *child*1 and *child*2). The parents are randomly chosen. Their genetic material is combined by a random one-point cross in the genes *G*_*g*_, conditions *C*_*c*_, and times *T*_*t*_ and mixing the coordinates in both children. We can see this process in Figure 6.

#### 3.4.8. Mutation

An individual can be mutated according to a probability of mutation, Mut. The mutation probability is verified for every individual and if it is satisfied, one out of six possible actions is taken. These actions are as follows: add a new random gene to *G*_*g*_ in TC, add a new condition to *C*_*c*_ in TC, or add a new time to *T*_*t*_ in TC or by removing a random gene, condition, or time. The election of these actions is also random. For the case of addition of a new gene, condition, or time, the operator checks whether the new member is already in the individual or not.

## 4. Results

We have applied the proposed measure MSR_3D_ as part of the TriGen algorithm to analyse several datasets: synthetically generated data, data from experiments with the yeast cell cycle (*Saccharomyces cerevisiae*) obtained from the Stanford University [16], three datasets retrieved from Gene Expression Omnibus [17], and a database repository of high throughput gene expression data. Two datasets are experiments for mouse (*Mus musculus*) [32, 33] and the third one is an experiment for humans (*Homo sapiens*) [34]. All experiments examine the behaviour of genes under conditions at certain times.

To examine the quality of the results in experiments with real datasets, we show for each experiment two types of validity measures: analysis of correlation among the genes, conditions, and times in each tricluster and analysis of genes and gene product annotations for the genes in each tricluster based on the Gene Ontology project [20].

Regarding the correlation analysis, we show a table for each tricluster (in rows) in which we calculate the Pearson and Filon [18] and Spearman [19] correlation coefficient between each combination of condition time and the values series are the expression levels of all genes in the corresponding condition-time combination. For example, for a tricluster with ten genes {1,…, 10}, three conditions {1, 3 and 5}, and two times {2 and 7}, we provide Pearson's and Spearman's correlation coefficient for values at the six possible combinations *V*_*c*=1,*t*=2_, *V*_*c*=1,*t*=7_, *V*_*c*=3,*t*=2_, *V*_*c*=3,*t*=7_, *V*_*c*=5,*t*=2_, and *V*_*c*=5,*t*=7_ for each of the ten genes.

In the biological analysis we provide a validation of the triclusters obtained based on the Gene Ontology project (GO) [20]. GO is a major bioinformatic initiative with the aim of standardizing the representation of gene and gene product attributes across species and databases. The project provides an ontology of terms for describing gene product characteristics and gene product annotation data. The ontology covers three domains: cellular component, the parts of a cell or its extracellular environment; molecular function, the elemental activities of a gene product at the molecular level such as binding or catalysis; and biological process, operations, or sets of molecular events with a defined beginning and end, pertinent to the functioning of integrated living units: cells, tissues, organs, and organisms. For legibility reasons, we have presented for one solution of the experiment a GO analysis table in which we include the most representative terms extracted by the Ontologizer software [35].

We have also provided a graphical representation of the triclusters found. For legibility reasons we show graphs for one tricluster for each of the experiments. Each tricluster is represented through three graphical views in which we can see the pattern of behavior. In the first (sample curves), we show one graph for each time, genes on the* x*-axis, the expression levels on the* y*-axis, and the lines of condition as the outline. In the second (time curves), we show for each experimental condition (one graph for each condition) genes on the* x*-axis, the expression levels in the* y*-axis, and the time lines as the outline. In the third representation (gene curves), for each experimental condition (one graph for each condition) we show times in the* x*-axis, the expression levels in the* y*-axis, and the genes as the outline.

All experiments were executed on a multiprocessor machine with 64 processors Intel Xeon E7-4820 2.00 GHz with 8 GB RAM memory. We have used Java for the TriGen algorithm implementation (and other ad hoc developments) and an R framework to create graphics and get datasets resources from GEO [17].

We now analyse the results obtained in each of the five experiments.

### 4.1. Synthetic Datasets

Synthetic data has the advantage that the process that generated the data is well known and so one is able to judge the success or failure of the algorithm [36]. Synthetic datasets generation has been widely applied both in microarray related publications [37, 38] and in other general data mining applications [39].

We have used an application designed by ourselves to generate the synthetic data applied in this work. The data generated is a three-dimensional dataset Dsynt_3D_ with 4000 genes (rows), 30 conditions (columns), and 20 times (depth) of random numbers generated by a cryptographic secure standard library Math3 provided by Apache Commons [40] where we insert 10 triclusters *T*Cale_*i*_, *i* ∈ 1,…, 10 with 3D patterns of 150 genes (rows), 6 conditions (columns), and 4 times (depth) at random positions within Dsynt_3D_.

To see the behavior of the MSR_3D_ measure applied along with TriGen and also with the aim of analyzing the effect of the value of the parameters in the solutions, we have made executions varying the number of solutions *N* in {100, 200} and other control parameters as follows.

- Number of generations *G* in {50, 100}: greater number of generations gives us an increase in genetic recombination of individuals; an excessive increase in *G* may favour exploitation versus exploration in excess and the algorithm may return solutions which fall into a local minimum.
- Number of individuals *I* in {300, 500}: an increase in the number of individuals creates a larger search space for the solutions; an excessive increase can create a scatter search effect and therefore not return good quality solutions.
- Rate of selection *Sel* in {0.5, 0.7, 0.9}: a high selection rate creates individuals with low level of genetic recombination, favouring exploitation versus exploration and if the parameter is increased in excess, the algorithm may fall into a local minimum.
- Probability of mutation *Mut* in {0.1, 0.5}: the opposite to the rate of selection. A high probability of mutation favours exploration versus exploitation, and if increased in excess you will end up with solutions in many areas of the search space but with low quality levels.
- Randomness in the initial population *Ale* in {0.5, 0.9}: increasing this parameter involves increasing the level of randomness in the initial population. This has to be combined with the overlap control to make sure that a wide area of the space of solutions is initially covered.
- Weight for the number of genes in the solution *w*_*g*_ in {0.0, 0.4}, weight for the number of conditions *w*_*c*_ in {0.0, 0.1}, and weight for the number of times *w*_*t*_ in {0.0, 0.1} control the number of items in the solutions; increasing these weights involves favouring solutions with more volume.
- Overlap control weights for genes, *wo*_*g*_ in {0.4, 0.7}, conditions *wo*_*c*_ in {0.0, 0.1}, and times *wo*_*t*_ in {0.0, 0.1}: the increase in these weights leads to little or nonoverlapped solutions; an excessive increase can lead us to lose interesting solutions.

The results obtained are shown in Table 2. We can see the high rate of coverage (90–96%) of the 10 different triclusters TCale_*i*_ inserted at random positions in the dataset Dsynt_3D_.

We can conclude that the MSR_3d_ measure applied along with TriGen algorithm was successful in finding the solution triclusters.

### 4.2. Yeast Cell Cycle Dataset

We have applied the TriGen algorithm to the yeast (*Saccharomyces cerevisiae*) cell cycle problem [16]. The yeast cell cycle analysis project's goal is to identify all genes whose mRNA levels are regulated by the cell cycle. The resources used are public and available in http://genome-www.stanford.edu/cellcycle/. Here we can find information relative to gene expression values obtained from different experiments using microarrays. In particular, we have created a dataset Delu_3D_ from the elutriation experiment with 7744 genes, 13 experimental conditions, and 14 time points. Experimental conditions correspond to different statistical measures of the Cy3 and Cy5 channels while time points represent different moments of taking measures from 0 to 390 minutes.

The parameter configuration used for this experiment is shown in Table 3.

With this configuration we wanted to find solutions with a considerable number of genes (*w*_*g*_ = 0.7) because it is the largest dimension on Delu_3D_. With the overlap control values we seek a compromise between slightly overlapped solutions and not losing interesting triclusters. The rest of the parameters have been set to a default configuration.

**Figure 7:**
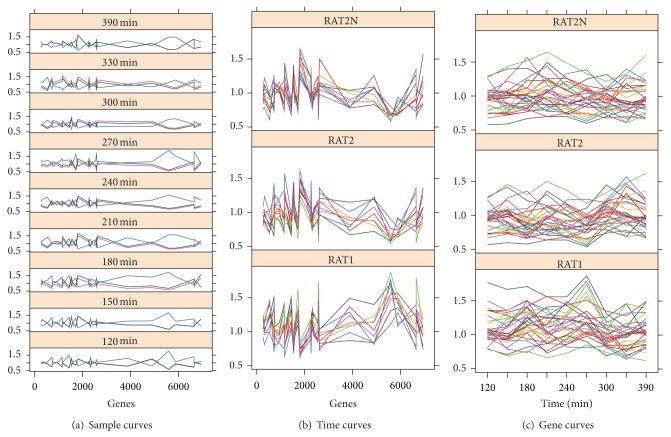
Graphical representation for tricluster TC_9_ found in the Yeast Cell Cycle Dataset.

To analyse the results, we can see the correlation in Table 4. We see how the correlation levels vary from very low up to almost perfect correlation. This is due to the fact that MSR_3D_ is capable of finding negatively correlated values, and some genes involved in the yeast cell cycle behave in an inversely correlated manner [41, 42] as can be seen in Figure 7(a). Therefore, when calculating the averages of correlations close to one and correlations close to minus one, we get values close to zero. Triclusters TC_8_, TC_15_, and TC_19_ stand out for having Pearson and Spearman correlation values close to one indicating an almost perfect correlation.

We also show a graphical representation of the genes, conditions, and times selected by tricluster TC_9_ with 30 genes, 3 conditions, and 9 time points in Figure 7. In Figure 7(a) we see a representation of genes at each condition with a graph for each time. The negative correlation among genes is clearly shown.  Figure 7(b) shows the genes at each time with one graph for each condition, and finally in Figure 7(c) we see the times at each gene with a graph for each condition.

In Table 5 we show an analysis of the biological annotations related to the genes selected in our tricluster TC_9_.

In this type of studies, *P* values are relevant below 0.05. We show the ten most significant terms with values ranking in the [0.001970,0.01039] interval. Furthermore, these terms are quiet specific increasing the quality of the tricluster obtained.

### 4.3. Mouse GDS4510 Dataset

This dataset was obtained from the GEO [17] with accession code GDS4510 and under the title* rd1 model of retinal degeneration: time course* [32]. In this experiment the degeneration of retinal cells in different individuals of home mouse (*Mus musculus*) is analyzed over 4 days just after birth, specifically on days 2, 4, 6, and 8. Our input dataset *DGDS*4510_3D_ is composed of 22690 genes, 8 experimental conditions (one for each individual involved in the biological experiment), and 4 time points.

We have executed the TriGen algorithm with the parameters shown in Table 6. We have increased the number of generations and individuals to create a larger search space as the input dataset has a considerable large volume. For the same reason we have increased *w*_*g*_ to favor individuals with a greater number of genes.

In Table 7 we see the correlation analysis for the 20 triclusters obtained. The correlation coefficients are very high and, in most cases, perfect with values close to one. This indicates almost perfect homogeneity between the genes, conditions, and times of the tricluster.

**Figure 8:**
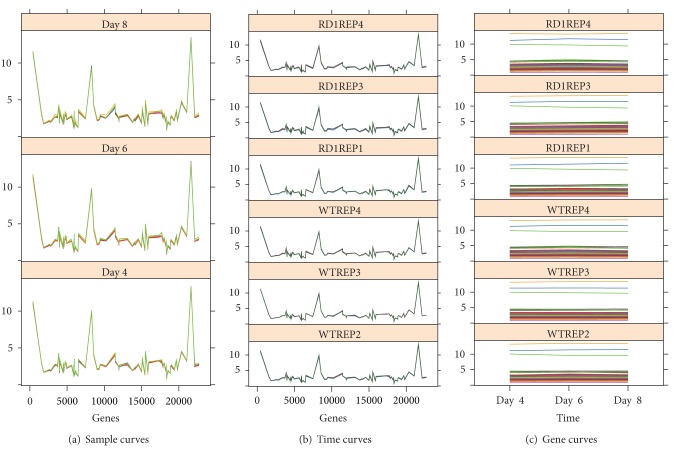
Graphical representation for tricluster TC_20_ found in the Mouse GSD4510 Dataset.

We show the graphs associated with solution TC_20_ with 78 genes, 6 conditions, and 3 time points in Figure 8. We see for the three views, Figures 8(a), 8(b), and 8(c), how all lines are totally aligned.

The biological validity of the solution shown can be found in Table 8 and yields good results regarding the terms listed and high statistical significance (*P* values below 0.05). The terms again are very specific and some are related to the dataset under study such as embryonic placenta development (GO:0001892) or cell differentiation involved in embryonic placenta development (GO:0060706).

### 4.4. Mouse GDS4442 Dataset

This time we have accessed the GEO database [17] to retrieve the dataset about the experiment under code GDS4442 titled* ectopic bHLH transcription factor expression Mesogenin1 effect on embryoid bodies: time course* [33]. This biological experiment examines the effect of doxycycline induction in mouse (*Mus musculus*) embryonic individuals at three stages of development: 12, 24, and 48 hours. Our input dataset *DGDS*4442_3D_ is composed by 45101 genes, 6 experimental conditions (one for each individual involved in the biological experiment), and 3 time points.

Regarding the TriGen parameters, we increased *G* and *I* for the same reason as in the previous experiment, that is, to have more solutions in the evolutionary process with a larger number of generations due to size of *DGSD*4442_3D_, see Table 9.

Regarding the correlation analysis, the results show high correlation values, highlighting the solutions TC_5_, TC_6_, and TC_15_ with Pearson's correlation values close to 1, see Table 10.

**Figure 9:**
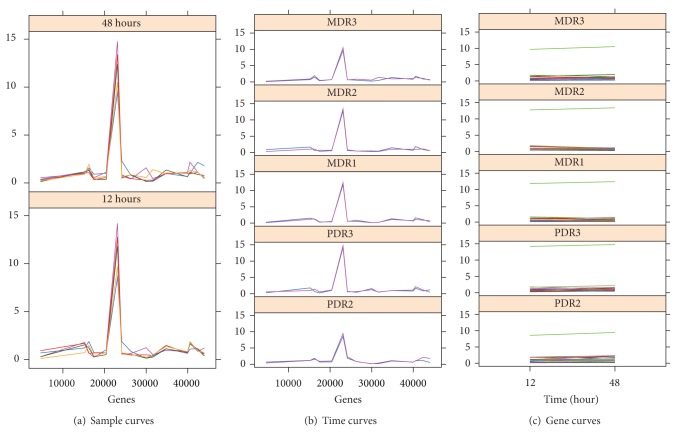
Graphical representation for TC_15_ found in the Mouse GSD4442 Dataset.

We show in Figure 9 the graphical representation of solution TC_15_ with 15 genes, 5 conditions, and 2 time points. We can see the great homogeneity among all genes, conditions, and times in Figures 9(a), 9(b), and 9(c).

The biological evaluation of tricluster TC_15_ shown in Table 11 shows annotated terms with high statistical significance, highlighting GO:0045127, GO:0009384, and GO:0019262 which are related to the cell wall synthesis which, in turn, is related to the action of doxycycline.

### 4.5. Human GDS4472 Dataset

This dataset has been obtained from GEO [17] under code GDS4472 titled* transcription factor oncogene OTX2 silencing effect on D425 medulloblastoma cell line: time course* [34]. In this experiment we analyze the effect of doxycycline on medulloblastoma cancerous cells at six times after induction: 0, 8, 16, 24, 48, and 96 hours. Our input dataset *DGSD*4472_3D_ is composed by 54675 genes, 4 conditions (one for each individual involved), and 6 time points (one per hour).

Because of the volume of the dataset *D*4472_3D_ we increase *G* and *I* to expand the space of solutions. The full set of parameters can be seen in Table 12.

We can see in Table 13 the high levels of correlation obtained for the 15 solutions found.

**Figure 10:**
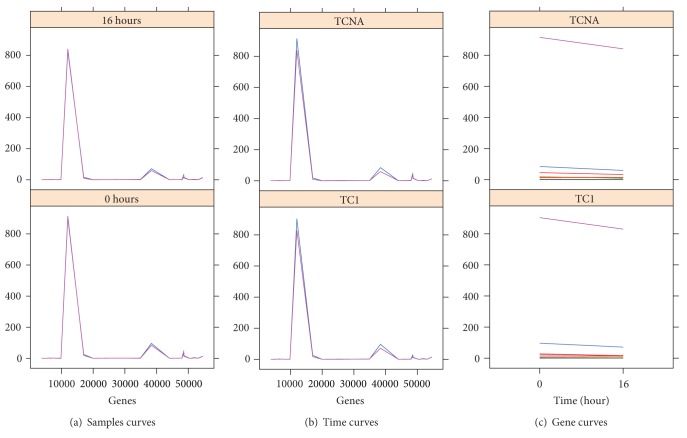
Graphical representation for tricluster TC_2_ found in the Human GDS4472 Dataset.

We graphically represent tricluster TC_2_ with 25 genes, 2 conditions, and 2 time points in Figure 10. We can see the great homogeneity among all genes, conditions, and times in Figures 10(a), 10(b), and 10(c).

The biological validation can be seen in Table 14, where we see annotated terms with high statistical significance.

## 5. Conclusions

In this work we have presented a new evaluation measure for triclusters, MSR_3D_, which measures the homogeneity among genes, conditions, and times in a tricluster. This measure has been inspired in the classic MSR measure proposed by Cheng and Church in [11]. A detailed formulation of both MSR and MSR_3D_ has been provided.

In order to assess the quality of the measure, we have applied it along with the TriGen algorithm [12], an evolutionary heuristic to mine triclusters from microarray experiments involving time, to several datasets: synthetically generated data, data from experiments with the yeast cell cycle (*Saccharomyces cerevisiae*) obtained from the Stanford University [16], and three datasets retrieved from Gene Expression Omnibus [17], two datasets are experiments for mouse (*Mus musculus*) and the third one is an experiment for humans (*Homo sapiens*). All experiments examine the behavior of genes under conditions at certain times.

The results obtained have been validated by means of analyzing the correlation among the genes, conditions, and times in each tricluster using two different correlation measures: Pearson and Filon [18] and Spearman [19]. Besides this, we have provided functional annotations for the genes extracted from the Gene Ontology project [20]. Regarding the synthetic data, we see that MSR_3D_ combined with TriGen has been capable of extracting almost all 10 triclusters artificially inserted in the dataset with a coverage of 90% to 96%. The results for the real datasets are also successful, with correlation values close to one, with the exception of the yeast dataset, where values are close to zero due to triclusters containing negatively correlated genes, found by MSR_3D_.

The GO validation has given good results as well, with high levels of significance for the terms extracted (*P* values smaller than 0.05 and very specific terms). Graphical representation of the triclusters has also been provided.

MSR_3D_ is a tricluster evaluation measure created to assess the quality of triclusters extracted from temporal experiments with microarrays, but it can be used in other biologically related fields, for instance combining expression data with gene regulation information by means of substituting the time dimension by ChIP-chip data representing transcription factor-gene interactions which can provide us with regulatory network information. This proposal can also be applied to mine RNA-seq data repositories. Triclustering can also be applied to not biologically related fields, for instance, the seismic regionalization of areas at risk of undergoing an earthquake [43]. In this case, the third component does not identify time points but features associated with every pair of geographical coordinates of the area under study.

## Figures and Tables

**Table 1:** TriGen algorithm parameters.

Parameter	Description
*N*	Number of triclusters extracted
*G*	Number of generations
*I*	Number of individuals in the population
Ale	Randomness rate
Sel	Selection rate
Mut	Mutation probability
*w*_*g*_	Weight for the number of genes
*w*_*c*_	Weight for the number of conditions
*w*_*t*_	Weight for the number of times
*wo*_*g*_	Weight for the overlap among genes
*wo*_*c*_	Weight for the overlap among conditions
*wo*_*t*_	Weight for the overlap among times

**Table 2:** TriGen algorithm synthetic match ratios.

Triduster	Match ratio
TCale_1_	91%
TCale_2_	91%
TCale_3_	90%
TCale_4_	96%
TCale_5_	95%
TCale_6_	95%
TCale_7_	95%
TCale_8_	95%
TCale_9_	95%
TCale_10_	95%

**Table 3:** TriGen algorithm control parameters for Yeast Cell Cycle Dataset.

Parameter	Values
*N*	20
*G*	200
*I*	50
Ale	0.3
Sel	0.5
Mut	0.3
*w*_*g*_	0.7
*w*_*c*_	0.5
*w*_*t*_	0.5
*wo*_*g*_	0.8
*wo*_*c*_	0.5
*wo*_*t*_	0.5

**Table 4:** Correlation results for tricluster Yeast Cell Cycle Dataset.

TC_sol_	Pearson	Spearman
1	−0.02	−0.02
2	0.32	0.28
3	0.17	0.24
4	0.37	0.37
5	0.02	0.02
6	0.05	0.04
7	0.03	0.03
8	0.99	0.98
9	0.04	0.03
10	0.03	0.01
11	0.03	0.03
12	0.02	0.02
13	0.06	0.03
14	0.04	0.04
15	0.99	0.98
16	0.03	0.01
17	0.01	0
18	0.03	0.03
19	0.92	0.9
20	0.03	0.02

**Table 5:** GO analysis for tricluster TC_9_ found in the Yeast Cell Cycle Dataset.

ID	Name	*P*-value
GO:0071012	Catalytic Step 1 spliceosome	0.001970
GO:0071006	U2-type catalytic Step 1 spliceosome	0.001970
GO:0072521	Purine-containing compound metabolic process	0.004610
GO:0051266	Sirohydrochlorin ferrochelatase activity	0.005208
GO:0004385	Guanylate kinase activity	0.005208
GO:0004747	Ribokinase activity	0.005208
GO:0006014	D-ribose metabolic process	0.005208
GO:0006986	Response to unfolded protein	0. 007288
GO:0046148	Pigment biosynthetic process	0.009738
GO:0070899	Mitochondrial tRNA wobble uridine modification	0.01039

**Table 6:** TriGen algorithm control parameters for Mouse GDS4510 Dataset.

Parameter	Values
*N*	20
*G*	500
*I*	300
Ale	0.4
Sel	0.4
Mut	0.2
*w*_*g*_	0.8
*w*_*c*_	0.3
*w*_*t*_	0.2
*wo*_*g*_	0.8
*wo*_*c*_	0.5
*wo*_*t*_	0.5

**Table 7:** Correlation results for tricluster Mouse GDS4510 Dataset.

TRI_sol_	Pearson	Spearman
1	1	0.99
2	1	1
3	1	1
4	1	1
5	1	1
6	1	1
7	1	1
8	1	0.99
9	1	0.99
10	1	1
11	1	0.99
12	1	1
13	1	0.99
14	1	1
15	1	1
16	1	1
17	0.99	0.99
18	1	1
19	1	1
20	1	0.99

**Table 8:** GO analysis for tricluster TC_20_ in Mouse GDS4510 Dataset.

ID	Name	*P*-value
GO:0004953	Icosanoid receptor activity	1.525 × 10^−6^
GO:0004955	Prostaglandin receptor activity	2.879 × 10^−5^
GO:0004954	Prostanoid receptor activity	3.729 × 10^−5^
GO:0001892	Embryonic placenta development	9.795 × 10^−5^
GO:0004958	Prostaglandin F receptor activity	1.595 × 10^−4^
GO:0060706	Cell differentiation involved in embryonic placenta development	2.868 × 10^−4^
GO:0001890	Placenta development	5.151 × 10^−4^
GO:0004982	N-formyl peptide receptor activity	7.342 × 10^−4^
GO:0009265	2′-deoxyribonucleotide biosynthetic process	7.342 × 10^−4^
G0:0046385	Deoxyribose phosphate biosynthetic process	7.342 × 10^−4^

**Table 9:** TriGen algorithm control parameters for Mouse GDS4442 Dataset.

Parameter	Values
*N*	15
*G*	500
*I*	400
Ale	0.4
Sel	0.5
Mut	0.4
*w*_*g*_	0.2
*w*_*c*_	0.8
*w*_*t*_	0.8
*wo*_*g*_	0.5
*wo*_*c*_	0.2
*wo*_*t*_	0.2

**Table 10:** Correlation results for tricluster Mouse GDS4442 Dataset.

TRl_sol_	Pearson	Spearman
1	0.52	0.53
2	0.34	0.36
3	0.49	0.53
4	0.52	0.45
5	0.92	0.79
6	0.86	0.83
7	0.39	0.31
8	0.35	0.44
9	0.46	0.44
10	0.6	0.58
11	0.62	0.62
12	0.59	0.58
13	0.76	0.61
14	0.61	0.64
15	0.98	0.6

**Table 11:** GO analysis for tricluster TC_15_ in Mouse GDS4442 Dataset.

ID	Name	*P*-value
GO:0045127	N-acetylglucosamine kinase activity	5.525 × 10^−4^
GO:0009384	N-acylmannosamine kinase activity	0.001105
GO:0019262	N-acetylneuraminate catabolic process	0.002208
GO:0004957	Prostaglandin E receptor activity	0.002760
GO:0006054	N-acetylneuraminate metabolic process	0.003862
GO:0050901	Leukocyte tethering or rolling	0.004412
GO:0051352	Negative regulation of ligase activity	0.004963
GO:0051444	Negative regulation of ubiquitin-protein ligase activity	0.004963
GO:0090136	Epithelial cell-cell adhesion	0.006063
GO:0001921	Positive regulation of receptor recycling	0.006612

**Table 12:** TriGen algorithm control parameters for Human GDS4472 Dataset.

Parameter	Values
*N*	15
*G*	500
*I*	300
Ale	0.2
Sel	0.3
Mut	0.4
*w*_*g*_	0.2
*w*_*c*_	0.5
*w*_*t*_	0.5
*wo*_*g*_	0.5
*wo*_*c*_	0.4
*wo*_*t*_	0.4

**Table 13:** Correlation results for Human GDS4472 Dataset.

TRI_sol_	Pearson	Spearman
1	0.94	0.78
2	1	0.8
3	0.86	0.87
4	0.98	0.72
5	0.98	0.81
6	0.95	0.93
7	0.99	0.67
8	0.99	0.62
9	0.88	0.71
10	0.88	0.73
11	0.99	0.75
12	0.85	0.69
13	0.89	0.72
14	1	0.76
15	0.98	0.67

**Table 14:** GO analysis for tricluster TC_2_ in Human GDS4472 Dataset.

ID	Name	*P*-value
GO:0002753	Cytoplasmic pattern recognition receptor signaling pathway	4.543 × 10^−4^
GO:2000299	Negative regulation of Rho-dependent protein serine/threonine kinase activity	8.415 × 10^−4^
GO:2000298	Regulation of Rho-dependent protein serine/threonine kinase activity	8.415 × 10^−4^
GO:2001264	Negative regulation of C–C chemokine binding	8.415 × 10^−4^
GO:2001263	Regulation of C–C chemokine binding	8.415 × 10^−4^
GO:0032479	Regulation of type I interferon production	0.001581
GO:0000226	Microtubule cytoskeleton organization	0.001755
GO:0032606	Type I interferon production	0.001762
GO:0070507	Regulation of microtubule cytoskeleton organization	0.001904
GO:0044092	Negative regulation of molecular function	0.001931
